# Microglial activation induces neuronal death in Chandipura virus infection

**DOI:** 10.1038/srep22544

**Published:** 2016-03-02

**Authors:** Abhishek Kumar Verma, Sourish Ghosh, Sreeparna Pradhan, Anirban Basu

**Affiliations:** 1 National Brain Research Centre, Manesar, Haryana-122051, India

## Abstract

Neurotropic viruses induce neurodegeneration either directly by activating host death domains or indirectly through host immune response pathways. Chandipura Virus (CHPV) belonging to family *Rhabdoviridae* is ranked among the emerging pathogens of the Indian subcontinent. Previously we have reported that CHPV induces neurodegeneration albeit the root cause of this degeneration is still an open question. In this study we explored the role of microglia following CHPV infection. Phenotypic analysis of microglia through lectin and Iba-1 staining indicated cells were in an activated state post CHPV infection in cortical region of the infected mouse brain. Cytokine Bead Array (CBA) analysis revealed comparatively higher cytokine and chemokine levels in the same region. Increased level of inducible nitric oxide synthase (iNOS), cyclooxygenase-2 (COX-2), Nitric Oxide (NO) and Reactive Oxygen species (ROS) in CHPV infected mouse brain indicated a strong inflammatory response to CHPV infection. Hence it was hypothesized through our analyses that this inflammatory response may stimulate the neuronal death following CHPV infection. In order to validate our hypothesis supernatant from CHPV infected microglial culture was used to infect neuronal cell line and primary neurons. This study confirmed the bystander killing of neurons due to activation of microglia post CHPV infection.

Inflammation as reported previously is a “double edged sword” for CNS^1^. Cases of chronic inflammation have been reported to cause tissue damage often leading to organ failures. In Central Nervous System (CNS) chronic inflammation results in neurodegeneration. Microglia are mesodermal cells of the brain that are activated under pathological conditions and tissue damage^2^,^3^. Transformation from resting to active state stimulates microglia to release various factors including cytokines, reactive oxygen species (ROS) and nitrogen species. Evidences have shown that although controlled level of release of these factors have a protective role, but an uncontrolled release of these factors in chronic conditions have a deleterious effect on neurons. Neurodegeneration is a common phenomenon that has been reported in cases of several neurotropic virus infections^4^,^5^,^6^. Under such condition of pathogenic attack microglia gets activated that subsequently leads to amplified secretion of the cytokines and chemokines that have been reported to kill the neurons in the vicinity of activation. This is presently termed as “bystander killing of neurons” where the neurons are forced to undergo apoptotic or necrotic death following external signalling from the microglia^7^,^8^,^9^,^10^,^11^. Bystander killing of neurons following viruses has been evidenced in various cases of neurotropic viral infections. But reports differ in their mechanistic view of this phenomenon.

CHPV was first isolated in the village of Chandipura, a small village in the district of Nagpur, Maharashtra, India while diagnosing people suffering from an unknown fever. Initially the researchers were suspecting the cause to be dengue or chikungunya virus but later it was identified to be belonging to the family of *Rhabdoviridae*^12^. Since the patients were not dying due to this virus infection researchers were reluctant to investigate on this virus. Moreover, there was no direct evidence that proved the virus was potent to cause symptomatic effect in the human patients. But with the sudden outbreak of epidemic in the Indian subcontinent in the year 2003, researchers started to investigate the causal pathogen. It was found as a result of several researches that CHPV has presently distanced itself from its sibling Vesiculovirus (which also does not have any human case reported) and has resulted in the massive outbreak^13^. Chandipura virus (CHPV) is a (−) single-stranded RNA virus. *Phlebotomus* sps. is the known vector for this virus^14^,^15^. Children under 15 years of age are more susceptible to this virus. CHPV has been reported to be an emerging human pathogen in the Indian subcontinent, with a case fatality of around 55 to 77%^16^,^17^,^18^,^19^. The symptoms are similar to other encephalitis attacks as characterized by acute fever, altered sensorium, seizures, diarrhoea, and vomiting. It has been reported previously that mouse dies post CHPV infection within 72–96 hours^20^. CHPV genome length is of 11 kb, comprised of 5 genes which encode a glycoprotein (G), a matrix protein (M), a nucleoprotein (N), a phosphoprotein (P protein), and a large polymerase protein (L).

For the past decade this virus is under the radar of investigation and researchers have gathered much information regarding its mode of replication and about its genome. But the host response and the neuropathogenesis of this virus was still an untold story. Neurons are safe place for replication and maturation of CHPV and it has been reported from our lab that this virus is not infecting astrocytes and microglia^21^. In this report we have explored the phenomenon of microglial activation pattern due to CHPV infection and measured the amount of pro-inflammatory cytokines secreted in different regions of the mouse brain post infection. According to previous studies reported from our lab CHPV induced direct killing of the neurons post infection through extrinsic apoptotic pathway^20^. But the source of the induction of apoptotic pathway is still unknown. In order to find an answer to this we hypothesized that microglial activation may have some pivotal role to play by bringing about the bystander killing of the neurons post CHPV infection.

## Results

### Evaluation of neurodegeneration in various brain regions post CHPV infection

**Figure 1:**
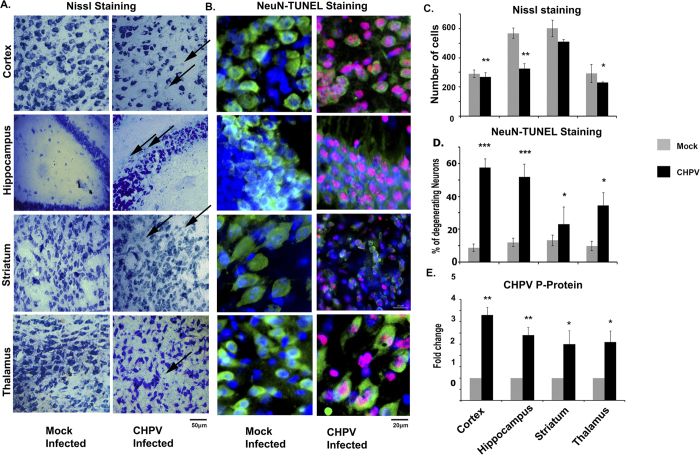
Evaluation of neuronal death post CHPV infection across the various brain regions. (**A**) Represents distribution of neuron in different brain regions. Dead cells are lightly stained or unstained indicated by arrow heads. (**B**) Co-expression of neuronal marker (NeuN) and cell death marker (TUNEL) represents the neurons undergoing apoptosis. (**C**) Representative graph shows analysis of cell counted for Nissl stain from an equivalent area from separate sections. (**D**) Representative graph shows percentage of neurodegeneration in different brain region. Calculated as: Percentage of dead cells = (Number of cells co-expressing NeuN and TUNEL)/Number of cells expressing NeuN) ×100. (**E**) Representative graph demonstrates qPCR mRNA level of viral P-Protein from different brain regions. *p < 0.05,**p < 0.01 and ***p < 0.005.

Mouse brain sections from cortex, hippocampus, thalamus and striatum were stained with Nissl for analysing the neuronal population post CHPV infection. Nissl positive cells from corresponding areas of CHPV infected tissue were counted and compared with that of the mock infected sections. Counting was done from 5 separate sections (both CHPV and mock infected) from 3 independent experiments. Analysis showed significant reduction in neuronal population in cortical and hippocampal regions compared to mock infected sections (Fig. 1AC). Further double staining of NeuN with TUNEL showed comparatively higher NeuN-TUNEL positive cells in the cortical and hippocampal regions when compared to other regions (Fig. 1BD). NeuN-TUNEL positive cell population was found to be least in striatal areas. Neun-TUNEL positive cells represent neuronal death. Since the neuronal death was found to be occurring differentially in various brain regions as per the Nissl and TUNEL staining results, we were interested to know whether the neurodegeneration was corresponding to a certain pattern of infection of CHPV in the brain. Quantitative PCR (qPCR) was performed to analyse the viral infection in different brain regions. Fig.1E indicated that there is no significant difference in viral replication irrespective of brain regions. Hence at this point we can state that CHPV infected and replicated in the brain without any bias to a particular region of the brain although there is a significant difference in neurodegeneration across the brain regions.

### Topographic distribution and activation pattern of Microglia following CHPV infection in brain

**Figure 2:**
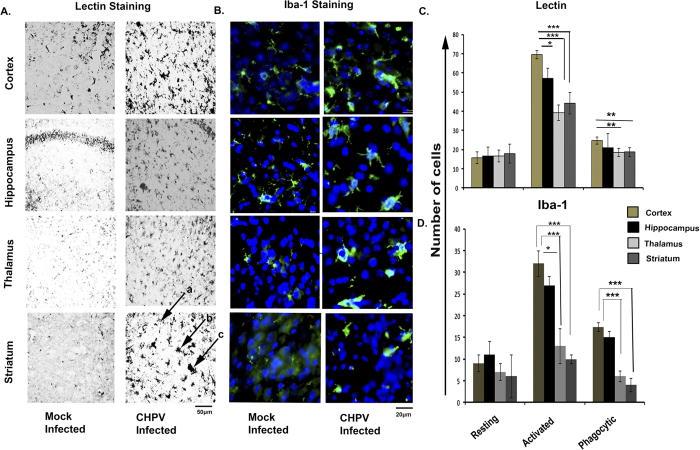
Topographic distribution and activation pattern of Microglia following CHPV infection in brain. (**A**) Lectin staining shows clear morphology of microglia in mock and CHPV infected brain tissue sample. Arrow heads a, b, and c shows resting, activated and phagocytic state respectively of microglia. (**B**) Iba-1 staining shows activated morphology of microglia which is greater in cortex and hippocampus when compared with thalamus and striatum. (**C**) Representative graph evaluates the number of microglia acquiring different morphological state after CHPV infection in different brain regions. (**D**) Representative graph shows quantitative analysis of resting and activated microglial morphology in different brain regions with Iba-1 staining. *p < 0.05 and ***p < 0.005.

Under the pathological conditions microglia undergo a series of stereotyped, morphological, phenotypic and functional changes in response to an activating stimulus. We examined the response of this resident immune cell of the CNS to CHPV infection in different brain regions. Lectin staining defines the microglial morphology. Using this histochemical stain, we have shown the presence of resting or quiescent cells exhibiting long thin rod like processes in mock infected animals. Following infection, the resting microglia decrease in number and were replaced by numerous “reactive microglial cells” with thicker processes and star-shaped morphology. Further microglia transformed from activated to amoeboid or phagocytic form by attaining rounded, darkly stained cell morphology. In the infected brain, the number of star-shaped “reactive” or “activated” microglia was more in the cortex compared to thalamus, striatum and hippocampus. These results indicated that CHPV infection is accompanied by robust microglial activation. We therefore determined the topographic distribution of resting, activated and phagocytic microglia in different brain region. 5 fields of lectin stained sections of infected brain were chosen for each region. Lightly stained rod-shaped cells were counted as resting cells, star-shaped cells as reactive cells and darkly stained round or triangular shaped cells were considered as amoeboid cells. Cells of each type were counted from five different sections and standard deviation across mean was calculated (Fig. 2A,C).

The result obtained from counting morphologically different cells showed that post CHPV infection resting cells have been transformed into activated morphology and the activated cells are maximum in cortex, hippocampus followed by striatum and thalamus. Lectin staining has shown the presence of amoeboid morphology that is significantly high in cortical and hippocampal regions (Fig. 2C). As a part of validation study tissue sections were further stained with Iba-1, a microglial marker. Different morphologies of microglia were obtained showing maximum activation in cortex followed by hippocampus (Fig. 2B). The activated microglia was least in striatum and thalamus. The representative graph compared number of activated microglia in mock infected and CHPV infected sections (Fig. 2D). A time dependent analysis of microglial activation was also observed by Iba-1 staining *in vivo* that showed an increasing number of activated microglia post CHPV infection (Fig. S1 A,B). Plaque assay analysis demonstrated that the microglial activation correlates with the propagation of CHPV in brain (Fig. S1C).

Hence Fig. 1 and 2 concomitantly proved that both cortex and hippocampus are affected by CHPV infection. These results prompted us to explore the probability of bystander killing of neurons by microglial activation.

### Proinflammatory cytokine and chemokine profile post CHPV infection

**Figure 3:**
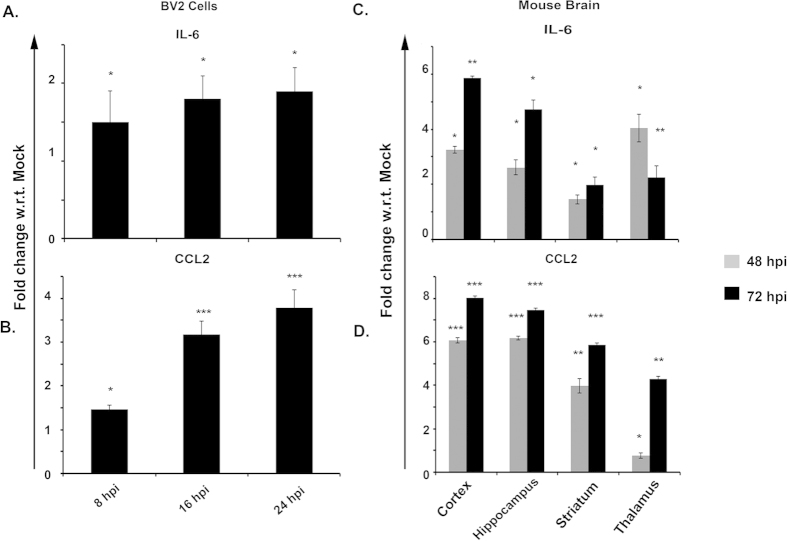
Topographic analysis of pro-inflammatory cytokines using CBA method. Protein from different brain region was isolated and inflammation was measured. (**A**) shows IL-6 level in BV2 lysate post CHPV infection at different time points. (**B**) CCL-2 level was measured in BV2 cells. (**C,D**) CBA was performed from brain lysate. IL-6 and CCL-2 was measured at 48 hpi and 72 hpi. *P < 0.05, **P < 0.01 and ***P < 0.0005.

Chronic activation of microglia stimulates release of cytokines and chemokines that have deleterious effect on neurons. We investigated the functional profile of activated microglia following CHPV infection in BV2 cells at 8, 16 and 24 hours post infection (hpi) as well as at 48 and 72 hpi from various regions of brain. CBA and qPCR analysis was performed to monitor the pro-inflammatory cytokine and chemokine levels in the BV2 lysate and brain homogenates (Fig. 3 and Fig. S2). CBA analysis showed significant enhancement of IL-6 level in BV2 cells at different time points and the highest fold change over control was at 24 hpi (Fig. 3A). In case of *in vivo*, a 6 & 5 fold change in expression level of IL-6 was observed in cortex and hippocampus, respectively at 72 hpi. Expression level of IL-6 showed an increasing trend in cortex, hippocampus and striatum in the course of CHPV infection except in case of thalamus (Fig. 3C). Similar to IL-6, CCL2 showed significant increase in its level over the time points in case of BV2 cells (Fig. 3B). CBA analysis indicated a robust increase in the level of CCL2 in cortex, hippocampus, thalamus as well as in striatum. The fold change over control for CCL2 was more in 72 hpi than at 48 hpi that was found to be consistent in all areas. Maximum fold change in CCL2 level was observed in cortex at both 48 as well as 72 hpi (Fig. 3D). A similar trend was observed in case of qPCR analysis (Fig. S2). A dose dependent study of cytokine level was conducted with different viral doses at 0.01 MOI, 1 MOI and 10 MOI and in presence of UV inactivated virus. No significant increase in IL-6 and CCL2 level was observed in case of UV inactivated virus (Fig. S3). Low viral dose was unable to elicit cytokine response whereas at higher dose IL-6 and CCL2 level showed an increasing trend over the time points. Interferon level was measured using ELISA from BV2 cell lines as well as from Brain lysate. We didn’t find significant difference in IFN-β and IFN-γ levels in supernatant collected at 8 hpi and 16 hpi from CHPV infected cells when compared with their corresponding mock infected samples. 1.4 fold change in IFN-β and 1.47 fold change in IFN-γ level was found at 24 hpi supernatant that was found to be significantly high (Fig. S4 A,B). Similarly ELISA for IFN-β from whole brain lysate showed 1.4 and 2.2 fold change at 48 hpi and 72 hpi, respectively, when compared with mock infected sample (Fig. S4C). IFN-γ also increased at later time points of infection when compared with mock infected samples (Fig. S4D).

### Induction of iNOS and COX-2 following CHPV infection

**Figure 4:**
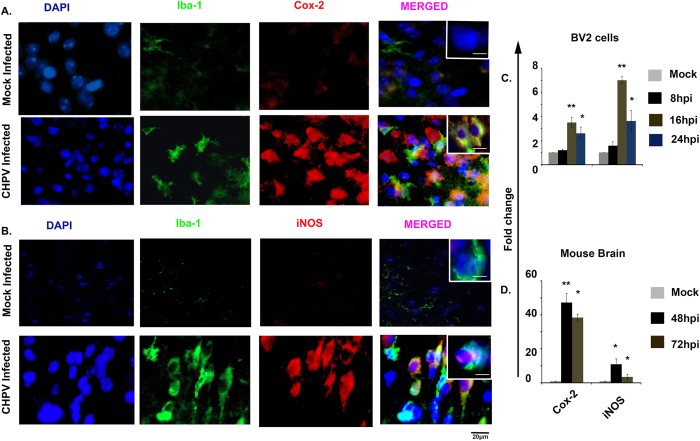
CHPV infection induces iNOS and COX-2 expression in brain. (**A**) Immunohistochemitry analysis of COX-2 and Iba-1 from mice brains after CHPV infection. Images represents COX-2 increased expression when compared with mock infected. Image in inset is of 63× magnification depicts colocolisation of COX-2 with Iba-1. (**B**) Immunohistochemitry images showing increased expression of iNOS from brain sections as compared to mock infected. Inset image is showing colocolisation of iNOS with Iba-1. mRNA level of COX-2 and iNOS was quantified using real time PCR. (**C**) Analysis of inducible nitric oxide synthase (iNOS), cyclooxygenase-2 (COX-2) from BV2 cells. (**D**) shows mRNA level in mouse brain at 48 hpi and 72 hpi. Values were normalized to GAPDH. *p < 0.05 and **p < 0.01.

Inducible nitric oxide synthase (iNOS) produces a large amount of nitric oxide (NO) in response to inflammatory mediators. We compared the mRNA levels of iNOS in BV2 cells at 8, 16 and 24 hpi and mouse brains at 48 and 72 hpi with mock infected. We performed immunohistochemistry to detect the expression of COX-2 and iNOS in mock infected and CHPV infected mouse brain sections. Expression of COX-2 increased following infection. Co-localization of COX-2 and Iba-1 in infected animals established that activated microglia released COX-2 following CHPV infection (Fig. 4A). Double staining using Iba-1 and iNOS antibody showed significantly higher number of double positive cells in infected sections compared to the mock infected sections (Fig. 4B). qPCR analysis indicated a 47 and 38 fold increase in the level of COX-2 while a 6 and 2 fold increase in the expression of iNOS was observed at 48 and 72 hpi, respectively when compared with their corresponding mock infected samples (Fig. 4CD).

### Measurement of ROS and NO secreted from CHPV microglial cells

**Figure 5:**
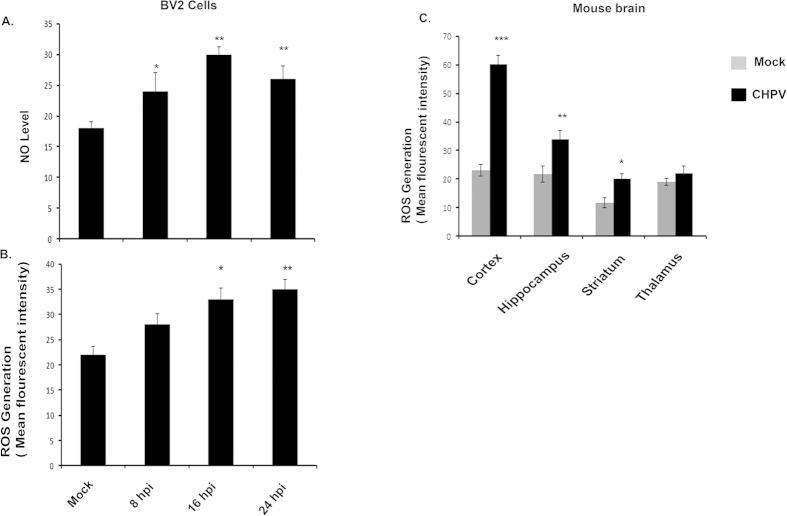
Generation of Nitric oxide (NO) and Reactive oxygen species (ROS). (**A**) NO generation was measured from BV2 cells using Griess reagent. The amount of nitrite accumulated was calculated (in μM) from a standard curve constructed with different concentrations of sodium nitrite. (**B**) Representative graph shows ROS level in BV2 cells. Level of ROS was maximum at 24 hpi followed by 16 hpi. (**C)** Plot showing intracellular ROS production, after different time of CHPV infection in Brain sample cells were collected from different regions and estimated for ROS generation. *p < 0.05, **p < 0.01 and ***p < 0.005.

Generation of reactive oxygen species (ROS) and nitric oxide (NO) by microglial cells in response to infection is a key marker for oxidative stress in these cells. We therefore measured level of ROS and NO from BV2 cells (Fig. 5AB). ROS from different regions of brain as previously mentioned was also analyzed (Fig. 5C). Both ROS and NO levels were checked at different incubation time points and were found to increase consistently with time. Level of NO was found to be significantly higher at 16 hpi that decreased further (Fig. 5A). ROS level in CHPV infected sample was significantly higher in 24 hpi compared to mock infected ones (Fig. 5B). ROS measurement from tissue lysate showed significantly higher level of ROS generation from cortex and hippocampus regions.

### Bystander neuronal killing by microglial activation

**Figure 6:**
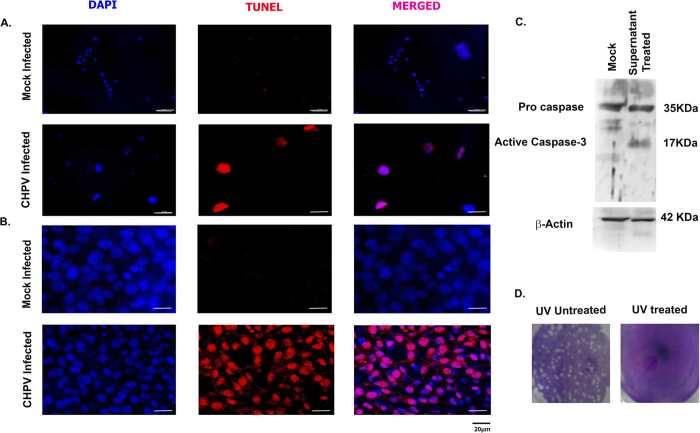
Bystander killing of neurons using supernatants from activated microglial cells. (**A**) *In vitro* cultured mouse cortical neurons were treated with supernatant (UV treated) for 12 hours and TUNEL staining was performed. The cells were counterstained with DAPI to study the nuclear morphology. Top panel shows staining in untreated cells while the lower panel shows supernatant treated cells. (**B**) *In vitro* culture of HT-22 neuron showed TUNEL positive cells in supernatant treated panel confirming bystander killing of neurons. (**C**) Immunoblot image shows expression of caspase 3 in supernatant treated HT-22 cells. β-actin was used as loading control. (**D**) Plaque assay on vero cell line showing no plaque in UV treated supernatant when compared with untreated supernatant.

Finally, in order to validate the bystander killing of neurons by activated microglia, primary cortical neurons along with HT-22 cell lines were treated with UV-treated supernatant collected from BV-2 cells. UV-treatment as mentioned in the methods section removed viral contamination from the cell supernatant. Hence we made sure that there was no pathogenic agent in the supernatants to induce the neuronal killing. Immunocytochemistry was performed to assess cell death *in vitro* using TUNEL staining. Neuronal cells treated with supernatant obtained from CHPV infected microglia showed significantly higher number of TUNEL positive cells compared to mock infected cells (Fig. 6A). For further validation of our hypothesis similar experiment was conducted in HT-22 cell line. TUNEL staining was performed. TUNEL positive cells were significantly higher compared to mock infected cells (Fig. 6B). These analyses indicated that microglial supernatant contained pro-inflammatory mediators that had lethal effect on neurons. Further, to confirm apoptosis caspase-3 level was monitored in HT-22 cell using immunoblotting. Blot analysis showed cleavage of caspase-3 in supernatant treated cell lysate (Fig. 6C). Plaque assay was performed to confirm virus free supernatant showing no plaques in UV treated supernatant (Fig. 6D).

## Discussions

Neurodegeneration in case of Chandipura Virus (CHPV) infection has been a well-known fact for some time now. Previously, our lab reported that neuronal death post CHPV infection was brought about through Fas- mediated extrinsic apoptotic pathway^20^. But the trigger that stimulated the neurons to undergo apoptosis was unknown. Our present study focused on finding that trigger. With the initial studies as suggested by the Fig. 1 we observed a significant amount of neurons were undergoing cell death in the cortical and the hippocampal regions of the brain post CHPV infection. In order to tally the observed neurodegeneration in those specific brain regions with the infection of CHPV we observed that the virus infected all the regions of the brain without a bias. Further to explore this result, we monitored the microglial activation in the various brain regions as shown in Fig. 2. Earlier reports suggested that microglia are ubiquitous and numerous in the CNS but not uniformly distributed^22^,^23^. Microglial population density is more in the telencephalon, followed by diencephalon, mesencephalon and rhombencephalon^24^. Compared to white matter, grey matter consist of more microglial cells. However, within these major divisions there is much heterogeneity in terms of both cell number and membrane density. Fig. 2 shows more activated and phagocytic microglia in the cortex and hippocampal regions followed by striatum and thalamus. Activation pattern of microglia and its corresponding regions related to neurodegeneration motivated us to explore the microglial role in neuronal killing.

Bystander killing of neurons through microglial activation has been observed in various cases of neuropathogenesis through viral infection^7^,^25^,^26^,^27^. As the primary immune effector cells of the CNS, microglia responds to injury, inflammation or the presence of pathogens by transforming to the “activated” state. When activated, microglial cells undergo proliferation, chemotaxis, and morphological alteration and generate numerous mediators involved in the inflammatory and immune-modulatory response. These mediators not only coordinate tissue remodelling in response to damage, but also possess several means by which they protect neurons from infection. In case of viral infection the microglial activation often results in chronic inflammation leading to brain death^28^,^29^,^30^. Our study has demonstrated for the first time that CHPV infection activates microglia both morphologically and functionally, *in vivo*. CHPV infection leads to an elevated level of pro-inflammatory mediator secretion. Microglia are the predominant source of pro-inflammatory mediators, and the cytotoxins released from activated microglia in cases of chronic infection are instrumental in inducing neuronal death. Our findings therefore suggest that microglial activation may be an important contributory factor in the pathogenesis of CHPV. The set of mediators that are elevated following CHPV infection consists of pro-inflammatory cytokines that would normally be associated with the recruitment of inflammatory cells, to the site of infection. These cytokines could contribute to the disruption of the blood brain barrier (BBB) that facilitates additional dissemination of the virus within the CNS. Pro-inflammatory cytokines and chemokines released by the microglial activation process bind to specific receptors of neurons that initiate the apoptotic mechanism in the cells. In order to validate our findings, we analysed the cytokine and chemokine levels from the brain regions mentioned earlier (Fig. 3). Following CHPV infection, expression levels of CCL2 and IL-6 was found to increase that were previously implicated in playing positive roles in encephalitis^31^,^32^,^33^,^34^. A region specific study of CCL2 and IL-6 expression reiterated our initial findings. Cortex showed the highest release of CCL2 and IL-6 from both 48 and 72 hpi (hours post infection) compared to other regions. Moreover, we studied the kinetics of the release of both these pro-inflammatory elements and observed an increasing trend over the time points (Fig. S2 & S3). These results guided our study in search of other deleterious inflammatory molecules.

Several studies have suggested an important role of enzyme inducible nitrogen oxide synthase (iNOS) and cycloxygenese-2 (COX-2) in the progression of neuronal damage after exictotoxic and ischemic injury^35^,^36^. Our further investigation suggested CHPV infection in the brain was followed by encephalitis symptom with the over-expression of iNOS and COX-2 (Fig. 4). Analysis from BV 2 cells showed enhanced levels of iNOS and COX-2 at 16 hours of infection that later decreased at 24 hpi and was consistent with the *in vivo* data. The significant increase in iNOS level at 48 hpi followed by the decline in level (compared to control) at 72 hpi, prompted us to speculate the existence of a compensatory mechanism that attempts to reduce the level of inflammation. Generation of ROS with the accumulation of oxidative damage has been implicated in neurodegenerative diseases and in the degradation of nervous system function. The brain is particularly vulnerable to oxidative stress on account of its high reactive oxygen levels, the presence of polyunsaturated fatty acids, transition metals in ionic form and low amounts of antioxidants^37^. Earlier it was reported that aberrant production of NO leads to peroxy-nitrite production can irreversibly damage lipid and protein synthesis causing cell death^38^. NO enhances glutamate excito-toxicity. Elevated level of ROS and NO in our case helped us to conclude that ROS and NO are also contributing to neuronal damage (Fig. 5). Although release of these mediators is meant for protection from pathogens and other brain insults, but it is also evident that it has irreplaceable deleterious effect in chronic infections. Microglia induces death directly by releasing different cytokines and chemokines or indirectly by infiltrating monocytes and T cells. *In vitro* microglial cells get infected and release different pro-inflammatory mediators. We have shown that CHPV infected microglial supernatant (UV radiated to remove viral contamination) treatment of both hippocampal neuronal cell line and primary neuronal culture with supernatant from microglial activation resulted in neuronal death. Observations from Fig. 6 helped us to conclude that microglial activation was stimulating the bystander killing of neurons following CHPV infection. Thus microglial activation may be one of the triggering factors for the neuronal apoptosis.

In conclusion, the present study suggested an answer to the question as to what triggers the apoptotic machinery in the neurons post CHPV infection. It would be of our future interest to identify the specific pathway of the bystander killing of neurons post CHPV infection. Moreover the existence of other triggering factors for the neuronal killing upon CHPV infection cannot be ruled out at this stage. Further research in this area would help the researchers to reveal the mechanistic model of neuropathogenesis of CHPV.

## Materials and Method

### Ethics statement

All animal experiments were approved by the Institutional Animal and Ethics Committee of the National Brain Research Centre (approval no. NBRC/IAEC/2013/88). The animals were handled in strict accordance with good animal practice as defined by the Committee for the Purpose of Control and Supervision of Experiments on Animals, Ministry of Environment and Forestry (CPCSEA), Government of India.

### Virus and Cell

CHPV (strain No. 1653514) was isolated from a human patient in Nagpur, 2003; kindly provided by Dhrubajyoti Chatopadhayay, University of Calcutta. The virus was propagated in the Vero cell line^39^. The titer of virus was analyzed to be 3 × 10^9^ pfu/ml using plaque assay technique as mentioned later in this section. HT-22 (immortalized mouse hippocampal neuronal cell line was gifted by Dr. Shiv Kumar Sharma, National Brain Research Centre) cells were used for our experiment with prior permission from Dr. Dave Schubert of Salk Institute from whom these cells were originally obtained. We also used BV2 cells (mouse microglial cell line, obtained from Dr. Steve Levison, Rutgers New Jersey Medical School, USA) to study the microglial response post CHPV infection. Both the cell lines mentioned here were grown at 37 °C in Dulbecco’s modified eagle medium (DMEM) supplemented with 3.7%, Sodium bicarbonate(Sigma, USA), 10% FBS(Gibco, Thermo Fischer Scientific, USA) and penicillin-streptomycin(Sigma, USA).

### Animal treatment

10 days postnatal BALB/c mouse pups were divided into two groups having five pups irrespective of sex in each group (since it is known that the virus does not have a gender bias for infection). CHPV group was injected with 50 μl of virus (approx. 1.5 × 10^5^ pfu/ml), while the mock infected group was injected with an equivalent amount of PBS through the intra-peritoneal (i.p.) route. Animals were sacrificed between 72–96 hours post-infection on the development of encephalitic symptoms in the CHPV group. Brains were excised after trans-cardial perfusion with ice cold 1X PBS.

For immunohistochemistry (IHC) analyses tissues were fixed using 4% paraformaldehyde (PFA) at 4 °C overnight after the excision. The following day tissues were dipped in sugar solution for 36 hours until they got fully submerged in the solution. After that tissues were processed for cryo-sectioning.

Brain tissues that were excised for RNA and protein extraction after excision were stored at −80 °C till use.

### Infection and treatment of BV2cells

BV2 cells were cultured in 10% serum containing media and were seeded in 60 mm plate at the density of 5 × 10^5^. After 12 hours the media was replaced by serum free media to limit the growth rate of the cells so that a particular number of cells can be monitored for infection. Post 2 hours incubation BV2 cells were infected with CHPV at multiplicity of infection (MOI) 0.1 for 2 hours and then cells were washed twice with 1X PBS to remove non internalized virus present in media. Cells at various post infection time points were harvested and the culture supernatants were collected and stored at −80 °C.

HT22 cells were incubated with UV inactivated supernatant of BV2 cell culture for 12 hours. Cells were then processed for cell death assay and protein extraction.

### CHPV inactivation process

CHPV inactivation was carried out with a UV cross-linker (UVC 500, Hoefer scientific, USA) using short-wavelength UV radiation (UVC, 254 nm) at a distance of 5 cm for 25 minutes on ice as described earlier^40^. Inactivation of virus was verified by plaque assay for all three sets of treated supernatant which showed the absence of viral plaque formation in the UV treated culture supernatant (Fig. 6C).

### Plaque assay

Plaque assay was performed following previously published protocol^20^. Vero cells were cultured in 10% FBS containing DMEM and seeded in 6 well plates at the density of 4 × 10^4^ cells/well. After complete monolayer formation was achieved serum containing media was changed to serum free media and incubated for 2 hours to acclimatize the cells for serum starvation. Meanwhile serial dilution was prepared in serum free DMEM starting with a 1:10 dilution of the stock solution (by adding 100 μL of UV inactivated supernatant and UV untreated supernatant in 900 μL media). The stock solution was serially diluted using 10-fold dilutions. Each dilution was added to each well of Vero cells. Post two hour of incubation with the respective dilutions at 37 °C, supernatants were removed and washed twice with 1X PBS to avoid multiple infection cycles. 3 ml of agarose overlay (9 ml 2% agrose (Roche, Germany), 10 ml 2X Minimal essential media (Sigma, USA), 1 ml FBS (Gibco, Thermo Fisher Scientific, USA), 100 μL penicillin-streptomycin (Sigma, USA)) was then added to each well. Plate was kept at 4 °C for solidifying the overlay after which it was returned to 37 °C for incubation of 24 hours. 4% PFA was added post incubation period for fixation of the cells for further analysis. Subsequently overlay was removed and cells were stained with crystal violet and plaque was counted. The viral titers were expressed as PFU/ml, calculated as ((number of plaques per well) × (dilution))/(inoculum volume).

### Primary neuron culture

Primary neurons were cultured according to previously published protocol^20^,^41^. Cells were then plated with 3 × 10^3^cells/well in poly-D- lysine (Sigma, USA) coated 4 welled chamber slides. Cells were incubated with serum-free medium for 4 hour before treating with UV inactivated supernatant of BV2 cell culture for 12 hours. For experimental treatments, the resting medium was exchanged for DMEM with N2 and B27 supplements, 25 mM KCl, and antibiotics. Mitosis inhibitor arabinoside (2 × 10^−5 ^M) was used to inhibit astrocyte proliferation. Cells were treated with CHPV infected and uninfected BV2 supernatant (both UV treated) for 12 hour following a similar procedure for infecting HT-22 cells as mentioned previously. After incubation, cells were processed for TUNEL assay.

### RNA isolation and real time PCR (qPCR)

Mouse brain tissue and harvested BV2 cells were homogenized using trizol reagent as per manufacturers’ protocol (TRI reagent, Sigma, USA). For qPCR analyses, cDNA was synthesized using Advantage RT-for-PCR kit (Clontech laboratories, CA). Oligonucleotide primers specific for mouse P-Protein, iNOS, COX-2, CCL2, and IL-6 were used as mentioned in Table 1. Power SYBR Green PCR master mix (Applied Biosystems) was used for the experiment. The qPCR results were analysed as per the user manual guidelines.

### Cytokine Bead Assay

The CBA kit (BD Biosciences, NJ, USA) was used to quantitatively measure cytokine levels in the mock-infected control and CHPV infected mouse brain samples. Using 50 μl of mouse inflammation standard and sample dilutions, the assay was performed according to the manufacturers’ instructions and analysed on the FACS Calibur (Becton Dickinson, CA, USA). Similar protocol was followed to analyze the cytokine levels for BV2 cells post CHPV infection.

### Lectin Staining

Post fixation of brain tissues in 4% PFA they were subjected to cryo-sectioning. Five sections from each region *viz.* cortex, hippocampus, striatum and thalamus were stained by tomato lectin as described earlier^42^. Briefly, the cryostat sections from mock infected and CHPV infected animals were washed with PBS followed by quenching with 0.3% H_2_O_2_. The sections were then rinsed with PBS containing CaCl_2_ (Qualigens, Thermo Fisher Scientific, USA), MgCl_2_ (Merck, MA, USA), MnCl_2_ (Sigma, USA), and then blocked with 1% BSA for 1 h. Post treatment sections were incubated overnight in a humidified chamber at 4 °C with biotinylated-tomato Lectin (10 g/mL; Vector laboratories, USA) diluted in PBS containing CaCl2, MgCl2, MnCl2, and 0.1%Triton-X-100. Following incubation in Streptavidin HRP (used as the secondary antibody at 1:500; Vector Laboratories), the sections were further incubated in inactivated DAB for 5 minutes followed by incubation in activated DAB for 10 minutes. The sections were then dehydrated in graded alcohol and xylene and mounted with DPX (Merck, MA, USA).

### Nissl’s Staining

Five sections from each brain areas as mentioned in the previous section were stained for 8–10 minutes cresyl violet stain followed by immersing in differentiation solution (100% alcohol + 1:1 Diaxone) for 4–5 minutes. Then sections were dipped in Diaxone for 3–5 minutes twice and in xylene for 3–5 minutes twice. Slides were mounted with coverslips using DPX (Merck, MA, USA). Then slide was kept for drying for 24 hours and was observed under bright field microscope.

### Cell Counting

Equal area from every section was taken and cells were counted from it. Five sections were counted for each sample and average was calculated. Counting was done manually using ImageJ software.

### Immunohistochemistry

Iba-1 (1:500, wako, USA) staining was performed on different brain region sections. Anti-Rabbit Alexa fluor 488 (1:1000; Molecular Probes, Invitrogen, USA) was probed as the secondary antibody of Iba-1. Fluorescence immunohistochemistry was performed for Mouse anti-Iba-1(1: 300, Chemicon, USA) for activated microglia which were then double stained with rabbit anti-iNOS (1:1000, Chemicon, USA) and rabbit anti-COX-2 (1:100, Cell Signaling Tech., Boston, USA), respectively. The corresponding secondary antibodies were used goat anti-mouse Alexa Fluor 594 (1:1000; Molecular Probes, OR) for Iba-1, goat anti-rabbit Fluorescein Isothiocyanate (FITC, 1:200; Vector Laboratories, USA) for both iNOS and COX-2. Double staining with NeuN: sections were deparaffinized, subjected to heat-induced epitope retrieval and incubated with NeuN antibody. After NeuN detection with an Alexa 488-labelled secondary antibody, sections were processed for TUNEL staining. Cell nuclei were stained with DAPI.

### TUNEL assay

Mouse hippocampal cell line HT22 was plated at density of 10^5^ cells/well in 2-well chamber slide with serum containing media. Cells were incubated with UV inactivated BV2 supernatant as per infection paradigm mentioned previously. At 12 hours post infection period, cells were subjected to *In situ* Cell Death Detection Kit, TMR red as per the manufacturers’ guidelines (Roche, Germany). Primary Neuronal cells were processed using similar guidelines as mentioned.

### ELISA

ELISA was performed to check protein expression level of interferon. BV2 cells were cultured and seeded in 60 mm dish and then standard infection was followed as described earlier. Then supernatants at different time point was collected and ELISA was performed as protocol described. In short plate was coated with antibody (diluted in coating buffer to 1 μg/ml) using coating buffer and then incubated for overnight. Next day after blocking for an hours in blocking buffer (1% BSA in PBS) supernatant was added to each well (100 μl) and incubated at RT for 6 hours, followed by three PBST wash. Then secondary biotin antibody (Biolegend) was added in each well (100 μl diluted in blocking solution 1 μg/ml), and incubated at RT for 30 minutes followed by three PBST wash and then incubation with streptavidin (Vector laboratories) for 30 minutes at RT. Substrate (TMB solution, Vector laboratories) was added and was incubated for 20 minutes and then stop solution was added for 100 μl per well. Optical Density was measured at 450 nm.

### Immunoblotting

Supernatant treated HT-22 cells were harvested for obtaining total cellular extracts, and the protein isolation procedure and immunoblotting steps were performed according to standard procedure. After being blocked with 5% skimmed milk, the membranes were incubated with primary antibodies against cleaved caspase-3 (Abcam, USA), at 1:1,000 dilutions. After extensive washes with 0.1% PBS-Tween, blots were incubated with the Anti-Rabbit peroxidase-conjugated secondary antibodies (Vector Laboratories, USA). The blots were processed for development using chemiluminescence reagent (Millipore, USA). The images were captured and analyzed using the Chemigenius bioimaging system (Syngene, United Kingdom). β-actin antibody (Sigma, USA) at 1:10,000 dilution was used as loading control.

### ROS Measurement

Intracellular ROS generation in control and treated cells was assessed using the cell permeable, non-polar H_2_O_2_ sensitive dye 5-(and-6)-chlromethyl-2′, 7′- dichloro dihydro fluorescein diacetate (CM-H2DCFDA) (Sigma Aldrich, USA) as described previously^41^. The extent to which H_2_O_2_ is generated is defined as the extent of ROS generation. Briefly, BV-2 cells were mock and CHPV infected for different time point. This was further followed with incubation in serum free media for 3 hour. Upon treatment, the cells were further treated with H2DCFDA (5 μM) for 30 minutes at 37 °C. Cells were washed twice with 1 × PBS and fluorescent intensity of the cells was measured using Varioskan flash multimode plate reader. To detect ROS from whole brain lysate, fresh brain homogenate sample were taken and incubated with DCFDA (5 μM) for 30 minutes at room temperature.

### Nitric oxide (NO) assay

Nitric oxide released from brain homogenates following CHPV infection was assessed using Griess reagent as described previously. Briefly, 100 μL of Griess reagent (Sigma, St. Louis, USA) was added to 100 μL of brain homogenate and incubated in dark for 15 minutes. The intensity of the color developed was estimated at 540 nm with the help of a Benchmark plus 96-well ELISA plate reader (Biorad, CA, USA). The amount of nitrite accumulated was calculated (in μM) from a standard curve constructed with different concentrations of sodium nitrite^40^.

### Statistical analysis

Values were calculated as the means of n = 3 independent experiments taking into consideration both −ve and +ve standard deviation (SD). In case of *in vivo* experiments each experimental groups consisted of 5 animals. Data from both mock-infected and experimental samples were compared using one-tailed Student’s *t* test in Graph Pad Prism 5 software. Statistical significance was set at *p* values of < 0.05 for all analyses.

## Additional Information

**How to cite this article**: Verma, A. K. *et al*. Microglial activation induces neuronal death in Chandipura virus infection. *Sci. Rep.* **6**, 22544; doi: 10.1038/srep22544 (2016).

## Supplementary Material

Supplementary Information

## Figures and Tables

**Table 1:** Various primers with forward and reverse sequence used for experiment.

Name of Primers	Sequence of Primers
P-Protein	Forward: 5′-CACAGCTTGGAACCTTCTCC-3′ Reverse: 5′-TGACCGGGTTGAGGATTGGC-3′
COX-2	Forward: 5′-AAGGCCTCCATTGACCAG-3′ Reverse: 5′-TCTTACAGCTCAGTTGAACGC-3′
iNOS	Forward: 5′-CCCTTCCGAAGTTTCTGGCAGCAGC-3′ Reverse: 5′-GGCTGTCAGAGCCTCGTGGTGGCTTTGG-3′
CCL-2	Forward: 5′-ACCAAGCTCAAGAGAGAGGT-3′ Reverse: 5′-CTGGATTCACAGAGAGGGAA-3′
IL-6	Forward: 5′-GAGGATACCACTCCCAACAGACC-3′ Reverse: 5′-AAGTGCATCATCGTTGTTCATACA-3′
