# Supplementary material for: Microglial activation induces neuronal death in Chandipura virus infection
Source: Sci Rep. 2016 Mar 2;6:22544. doi: 10.1038/srep22544 (PMC4773833; doi:10.1038/srep22544)
Supplement: Supplementary Information [file srep22544-s1.pdf]

Microglial activation induces neuronal death in Chandipura virus infection

Abhishek Kumar Verma, Sourish Ghosh, Sreeparna Pradhan[¥], Anirban Basu[#]

National Brain Research Centre, Manesar, Haryana-122051, India

# To whom correspondence should be addressed:
Anirban Basu,
National Brain Research Centre,
Manesar, Haryana-122051, India.
Email: anirban@nbrc.ac.in

¥ McGill University, Montreal, Canada.

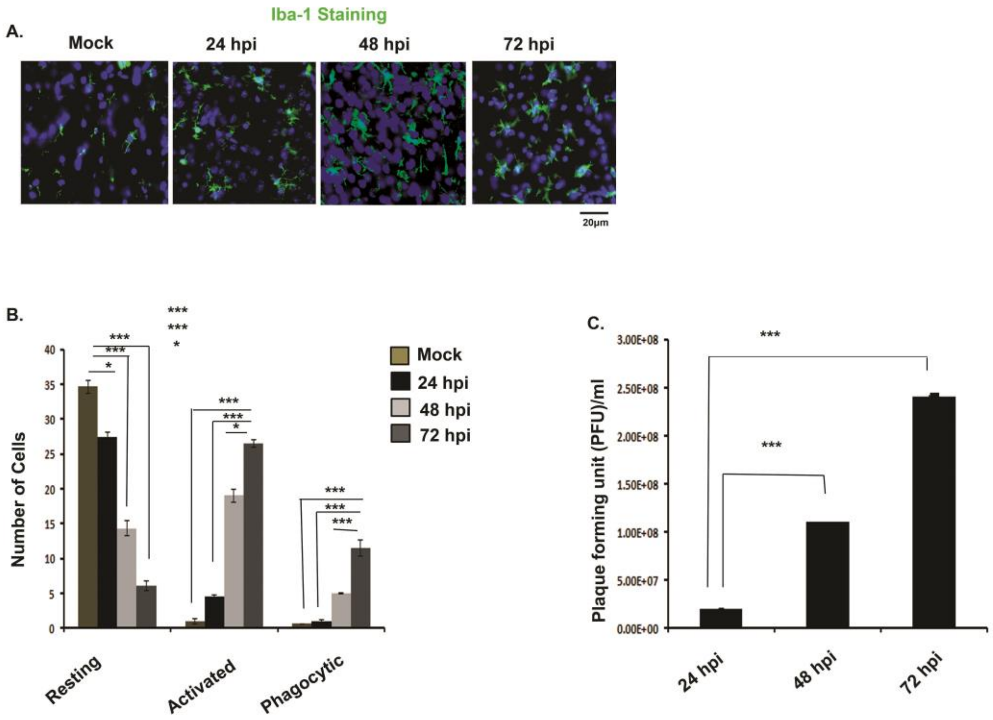

Fig S1 Iba-1 staining in brain section shows different microglial morphology throughout the brain. Different sections were stained with microglial marker Iba-1 and cell counting was done from five different sections from each time point. A.) shows Iba-1 and DAPI merged image at 24 hpi, 48 hpi, 72 hpi and compared it with mock infected animal. B.) Bar graph shows number of cells of different morphology at different time point. Resting cells are maximum in mock infected sections followed by 24hpi , 48 hpi and then 72 hpi. Maximum activation was found at 72 hpi and was least in mock infected sections. During onset of symptom, microglia has changed to phagocytic morphology which was maximum at 72 hpi. C.) Plaque assay from brain harvested at different time point shows increasing titer of CHPV as time progresses. Maximum number of plaque was found 72hpi.

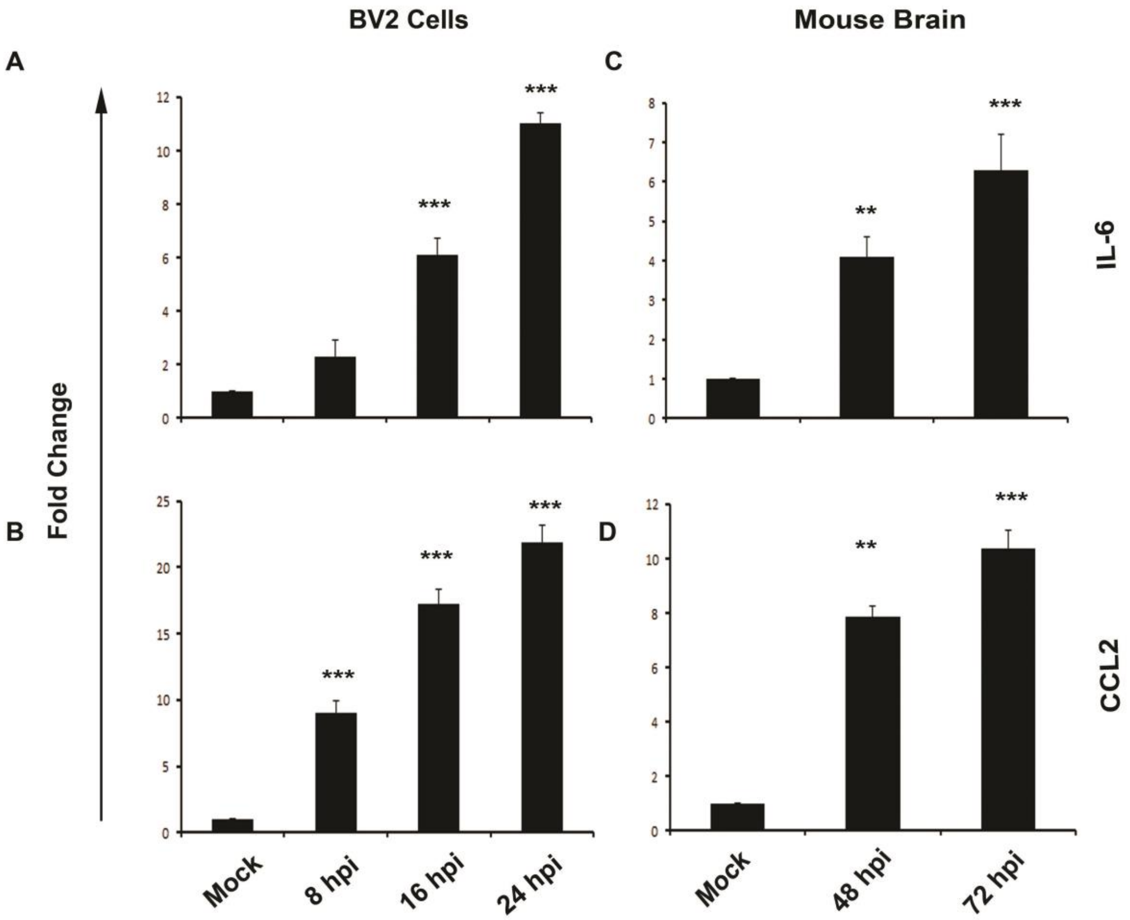

Fig S2. mRNA level for IL-6 and CCL2 was checked from BV2 cells and from infected brain at different time point. A.) Bar graph shows IL-6 level from BV2 cells in mock infected as well as cells harvested at 8hpi, 16hpi, 24hpi. Maximum mRNA level was found at 24hpi followed by 16 hpi and 8 hpi. B.) mRNA level for CCL2 was found maximum at 24hpi followed by 8hpi in BV2 cells. C.) IL-6 mRNA level from brain collected at 48hpi and 72 hpi shows maximum change at 72 hpi when compared with mock infected samples. D.) Shows CCL2 level from Brain and was found maximum at 72 hpi. (*** for p value < 0.005)

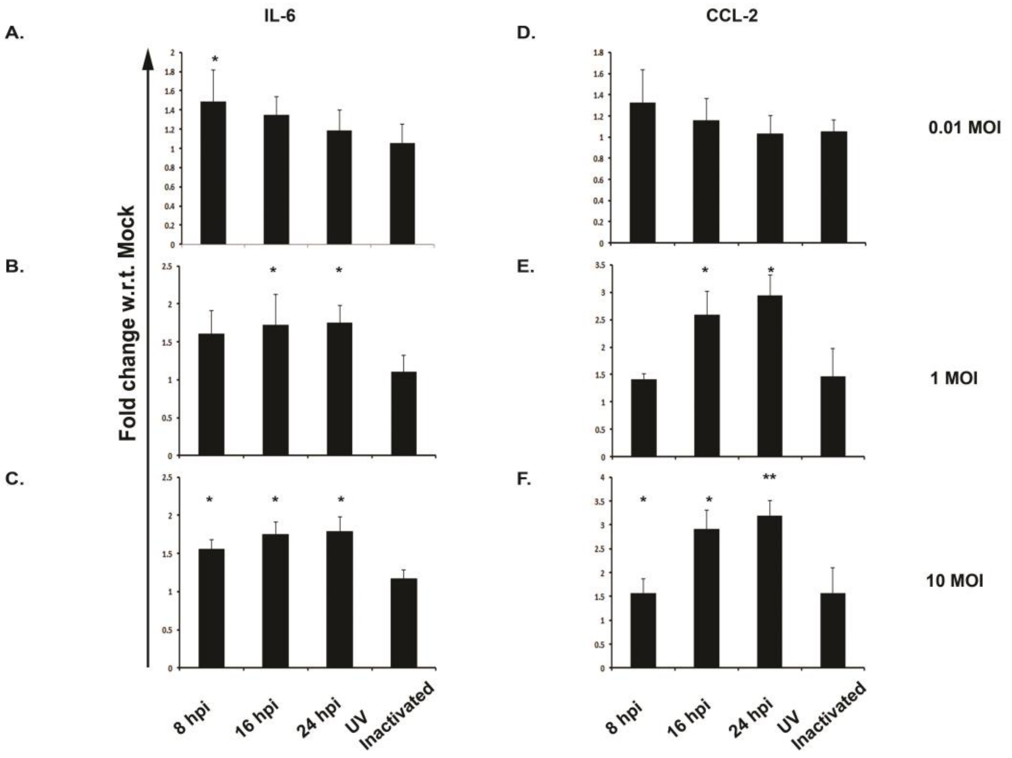

Fig S3. Cytokines level was measured at different viral dose as well as different time of infection. IL-6 and CCL2 level was measured from BV2 cells harvested at 8hpi, 16hpi, 24hpi and UV inactivated virus at 0.001 MOI, 1 MOI, 10 MOI. A,B &C shows level of IL-6 at differrnt dose of virus. 0.01 MOI of infection was unable to elicite cytokine response whereas at 1 MOI and 10 MOI of infection increased level of IL-6 was found. Maximum level was found at 24hpi followed by 16 hpi. D, E, &F shows CCL2 level measured in similar condition as IL-6. 0.01 MOI was unable to elicite CCL2 response whereas at higher viral dose increase was recorded. Maximum increase was found at 24 hpi. UV inactivated virus was unable to elicite cytokine response.

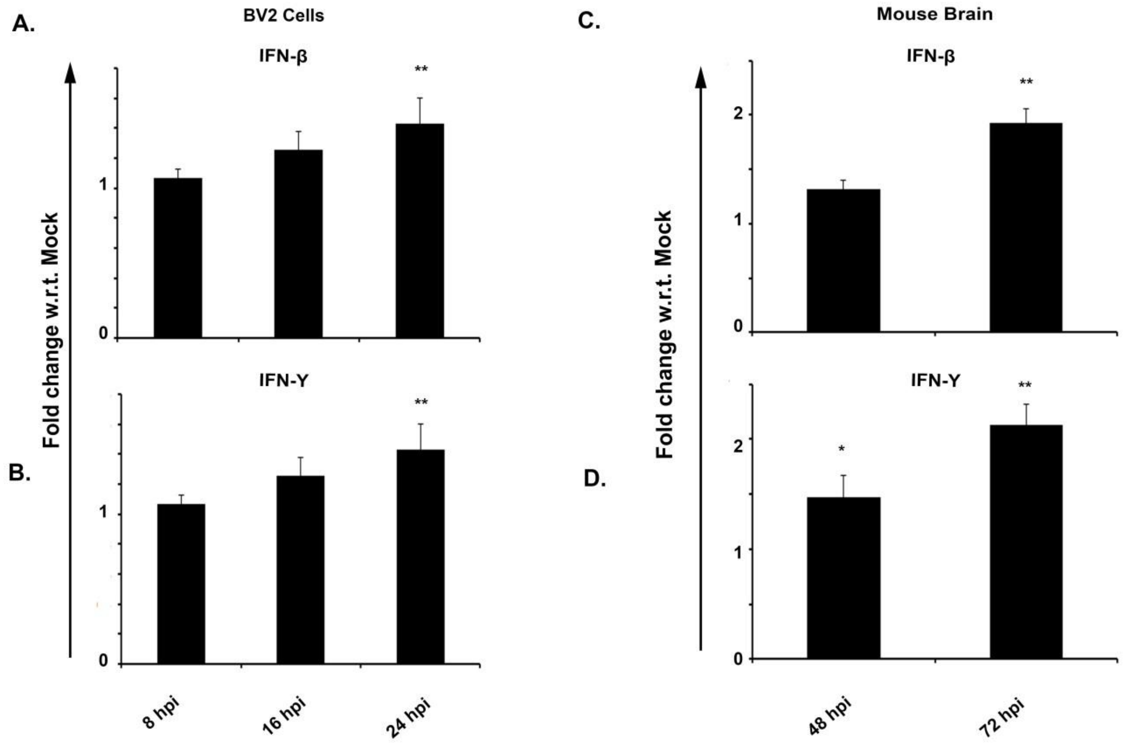

Fig S4. ELISA was performed to check protein expression level of Interferon. IFN-β and IFN-Y level was checked from both BV2 cell line as well as from Brain lysate. A. IFN-β level was checked from supernatant collected from mock as well as CHPV infected cells and then processed for ELISA. Significant fold change was found at 24 hpi of infection when compared with mock. B. IFN-Y level was checked from supernatant in similar condition as described earlier and 1.5 fold change was found at 24hpi. C. ELISA from whole Brain lysate for IFN-β shows 1.4 and 2.2 fold change at 48hpi and 72 hpi respectively . D. IFN-Y level was found to be 1.47 and 2.2 when compared with Mock infected samples. *P<0.05, **P<0.01 and ***P<0.0005.

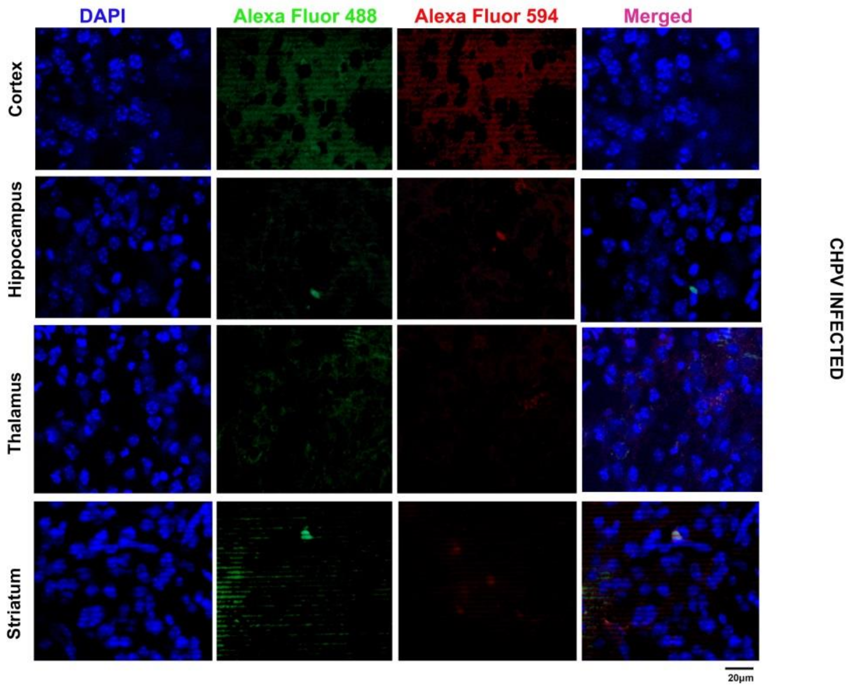

Fig S5. Negative control for IHC. We are showing it from different region of brain.
